# Protein Transfection Study Using Multicellular Tumor Spheroids of Human Hepatoma Huh-7 Cells

**DOI:** 10.1371/journal.pone.0082876

**Published:** 2013-12-05

**Authors:** Takuma Kato, Masakazu Tanaka, Makoto Oba

**Affiliations:** Graduate School of Biomedical Sciences, Nagasaki University, Nagasaki, Japan; Okayama University, Japan

## Abstract

Several protein transfection reagents are commercially available and are powerful tools for elucidating function of a protein in a cell. Here we described protein transfection studies of the commercially available reagents, Pro-DeliverIN, Xfect, and TuboFect, using Huh-7 multicellular tumor spheroid (MCTS) as a three-dimensional *in vitro* tumor model. A cellular uptake study using specific endocytosis inhibitors revealed that each reagent was internalized into Huh-7 MCTS by different mechanisms, which were the same as monolayer cultured Huh-7 cells. A certain amount of Pro-DeliverIN and Xfect was uptaken by Huh-7 cells through caveolae-mediated endocytosis, which may lead to transcytosis through the surface-first layered cells of MCTS. The results presented here will help in the choice and use of protein transfection reagents for evaluating anti-tumor therapeutic proteins against MCTS models.

## Introduction

Multicellular tumor spheroid (MCTS) is known to be a very useful three-dimensional *in vitro* tumor model, which represents the morphological and functional features of *in vivo* avascular solid tumors [1–3]. MCTS is characterized by actively proliferating outer cell layers and hypoxic and quiescent inner cells. Compared to monolayer cultured cells, a long-term culture can be achieved by spheroid cell cultures with the sufficient maintenance of their functions. Therefore, MCTS is a good experimental model located between an *in vitro* monolayer cultured cell model and *in vivo* animal model. This model has been widely used not only for screening ani-tumor drug candidates [4,5], but also for investigating drug delivery systems (DDS) [6–9]. The deep percolation of anti-tumor drugs and their DDS into tumor tissues is necessary for successful therapy, and this can be evaluated using MCTS models.

Proteins are one of the most important biomacromolecules in all living cells. The application of proteins to research has ranged from biochemical experiments to drug discoveries. Proteins are easily degraded by protease and deactivated in or out of cells. A major key for the success of delivering proteins to cells directed to biochemical and drug discovery studies is the development of protein delivery systems with high efficiency and negligible cytotoxicity [10,11]. Several protein transfection reagents are commercially available due to the extensive development of excellent delivery systems [12,13]. Their reagents are powerful tools for elucidating the function of a protein in a cell and controlling cellular functions by an introduced protein.

We recently reported the intracellular internalization mechanism of three different commercially available protein transfection reagents, the lipid-based Pro-DeliverIN, peptide-based Xfect, and cationic polymer-based TurboFect [14]. These reagents were internalized into monolayer cultured HeLa cells by different mechanisms, which may be helpful in choosing and using protein transfection reagents for experiments. To gain further information into the biological properties of these reagents, we reused Pro-DeliverIN, Xfect, and TurboFect in this study, and evaluated their complexes with bovine serum albumin (BSA) against human hepatoma Huh-7 MCTS models as well as monolayer cultured cell models. We have already reported that Huh-7 cells were good models for MCTS [8,9]. Less attention has been paid to studies on protein transfection reagents using MCTS models. Cellular uptake studies using specific inhibitors of endocytosis and confocal laser scanning microscope (CLSM) observations clarified the internalization routes and final localization of each complex in Huh-7 MCTS. The results obtained here may be informative for using protein transfection reagents against MCTS to screen and evaluate anti-tumor therapeutic proteins.

## Materials and Methods

### Materials

Pro-DeliverIN was purchased from OZ Biosciences (Marseille, France). Xfect was obtained from Clontech Laboratories, Inc. (Palo Alto, CA, USA). TurboFect was purchased from Fermentas (Glen Burnie, MD, USA). Dulbecco’s modified Eagle’s medium (DMEM), bovine serum albumin (BSA), fluorescein isothiocyanate conjugate BSA (FITC-BSA), filipin III from *Streptomyces filipinesis*, and amiloride hydrochloride were purchased from Sigma-Aldrich Co. (ST. Louis, MO, USA). Heparin, Cell lysis buffer M, and sucrose were the products of Wako Pure Chem. Co., Ltd. (Osaka, Japan). Hoechst 33342 was purchased from Dojindo Laboratories (Kumamoto, Japan). 

### Preparation of protein transfection reagent/BSA complex

Each protein transfection reagent/BSA or FITC-BSA complex was prepared according to the manufacture’s protocols and the previous study [14]. Briefly, 1.0 μL of Pro-DeliverIN reagent, 3.0 μL of 1X Xfect protein transfection reagent stock solution, and 0.8 μL of TurboFect protein transfection reagent were used to prepare complexes containing 1.0 μg of BSA or FITC-BSA, respectively.

### Dynamic light scattering (DLS) measurement

The size of BSA complexes was evaluated by DLS using Nano ZS (ZEN3600, Malvern Instruments, Ltd., UK). A He–Ne ion laser (633 nm) was used as the incident beam. The data obtained at a detection angle of 173° and a temperature of 37°C were analyzed by a cumulant method to obtain the hydrodynamic diameters and polydispersity index (PDI) (*μ*/Γ^2^) of complex. The results are presented as the mean and standard deviation obtained 3 measurements.

### Zeta-potential measurement

The zeta-potential of BSA complexes was evaluated by the laser-Doppler electrophoresis method using Nano ZS with a He–Ne ion laser (633 nm). The zeta-potential measurements were carried out at 37°C. A scattering angle of 173° was used in these measurements. The results are presented as the mean and standard deviation obtained 3 measurements.

### Cell culture and preparation of multicellular tumor spheroid (MCTS)

Human hepatoma Huh-7 cells (JCRB Cell Bank, Osaka, Japan) were maintained in DMEM supplemented with 10% fetal bovine serum in a humidified atmosphere containing 5% CO_2_ at 37°C. MCTS was prepared by using a 96-well culture plate designed for spheroid formation (Sumiloncelltight, Sumitomo Bakelite, Tokyo, Japan) as reported previously [8,9]. Briefly, 200 μL of the cell suspension (2,500 cells/mL) was seeded onto a 96-well culture plate. After overnight incubation, MCTS with a diameter of ca. 150 μm was spontaneously formed in each well. 

### Cellular uptake using monolayer cultured cells

Each protein transfection reagent/FITC-BSA complex was used for these experiments. Huh-7 cells were seeded onto 96-well culture plates (10,000 cells/well) and incubated overnight in 100 μL of medium. The medium was replaced by fresh medium and protein transfection reagent/FITC-BSA complex solution was then applied to each well (0.25 μg FITC-BSA/well). After 3h incubation, the medium was removed, and cells were washed 3 times with ice-cold PBS supplemented with heparin (20 units/mL) and treated with Cell lysis buffer M. The fluorescence intensity of the lysate at 515 nm (white LED excitation) was measured by a spectrofluorometer (ND-3300, NanoDrop, Wilmington, DE, USA) and cellular uptaken protein (dose%) was calculated using a calibration curve. The results are presented as the mean and standard deviation obtained from 4 samples.

### Inhibition of endocytosis using monolayer cultured cells

Huh-7 cells were seeded onto 96-well culture plates (10,000 cells/well) and incubated overnight in 100 μL of medium. After replacement with fresh medium in the absence or presence of sucrose (0.4 M), amiloride (5 mM), or filipin (5 μg/mL), cells were pre-incubated at 37°C for 30 min. Each protein transfection reagent/FITC-BSA complex solution was then applied to each well (0.25 μg FITC-BSA/well) and incubated for 1h (in the case of amiloride and filipin) or 3h (in the case of sucrose). The experimental conditions were as reported previously [14]. After each incubation time, the medium was removed and cells were washed 3 times with ice-cold PBS supplemented with heparin (20 units/mL) followed by treatment with Cell lysis buffer M. The fluorescence intensity of the lysate at 515 nm (white LED excitation) was measured by the ND-3300. The results are presented as the mean and standard deviation obtained from 3 samples.

### Cellular uptake using MCTS

Each protein transfection reagent/FITC-BSA complex solution was applied to each well with MCTS (0.25 μg FITC-BSA/well). After 3h incubation, the medium was removed, and MCTS was washed 3 times with ice-cold PBS supplemented with heparin (20 units/mL) followed by treatment with Cell lysis buffer M. The fluorescence intensity of the lysate was measured by a spectrofluorometer (ND-3300, NanoDrop) and cellular uptaken protein (dose%) was calculated using a calibration curve. The results are presented as the mean and standard deviation obtained from 4 samples.

### Inhibition of endocytosis using MCTS

MCTS was pre-incubated at 37°C for 30 min with medium in the absence or presence of sucrose (0.4 M), amiloride (5 mM), or filipin (5 μg/mL). Each protein transfection reagent/FITC-BSA complex solution was then applied to each well (0.25 μg FITC-BSA/well) and incubated for 1h (in the case of amiloride and filipin) or 3h (in the case of sucrose). After each incubation time, the medium was removed and cells were washed 3 times with ice-cold PBS supplemented with heparin (20 units/mL) followed by treatment with Cell lysis buffer M. The fluorescence intensity of the lysate at 515 nm (white LED excitation) was measured by the ND-3300. The results are presented as the mean and standard deviation obtained from 4 samples.

### Confocal laser scanning microscope (CLSM) observations using MCTS

Each protein transfection reagent/FITC-BSA complex solution was applied to each well with MCTS (0.25 μg FITC-BSA/well). After 3h incubation, the medium was removed, and spheroids were washed 3 times with ice-cold PBS supplemented with heparin (20 units/mL). In the case of inhibitory experiments (Figure S2), MCTS was pre-incubated at 37°C for 30 min with medium in the absence or presence of sucrose (0.4 M), amiloride (5 mM), or filipin (5 μg/mL). Each protein transfection reagent/FITC-BSA complex solution was then applied to each well (0.25 μg FITC-BSA/well) and incubated for 1h (in the case of amiloride and filipin) or 3h (in the case of sucrose). The intracellular distribution of the protein transfection reagent/FITC-BSA complex was observed by a confocal laser scanning microscope (CLSM) after staining nuclei with Hoechst 33342. CLSM observations were performed using an LSM 710 (Carl Zeiss, Oberlochen, Germany) with an EC Plan-Neofluar 40X/1.3 objective (Carl Zeiss) or Plan-Apochromat 100X/1.4 objective (Carl Zeiss) at an excitation wavelength of 405 nm (UV laser) and an emission wavelength of 409—484 nm for Hoechst 33342, and 488 nm (Ar laser) and 494—542 nm for FITC-BSA.

### Statistical analysis

Statistical significance was assessed by 2-tailed Student’s t-test. *P* values of less than 0.05 were considered significant.

## Results

### Physicochemical characterization of BSA complexes

The mean size and zeta-potential of BSA complexes were different according to the protein transfection reagents (Table 1). Xfect showed small size of 32.3 nm and positive charge of +8.89 mV, while Pro-DeliverIN and TuboFect showed bigger sizes of 832 and 393 nm and negative charges of –13.2 and –25.9 mV, respectively. PDI of Pro-DeliverIN, Xfect, and TurboFeect were 0.394, 0.342, and 0.551, which were quite high compared to ideal monodisperse nano particles.

**Table 1:** Size and zeta-potential of each protein tranfection reagent/BSA complex.

	**size(nm)**	**PDI(*μ*/Γ^2^)**	**zeta-potential(mV)**
**Pro-DeliverIN**	832±53	0.394±0.044	–13.2±4.3
**Xfect**	32.3±4.2	0.341±0.007	8.89±0.31
**TurboFect**	393±12	0.551±0.039	-25.9±1.0

### Cellular uptake of BSA complexes

**Figure 1:**
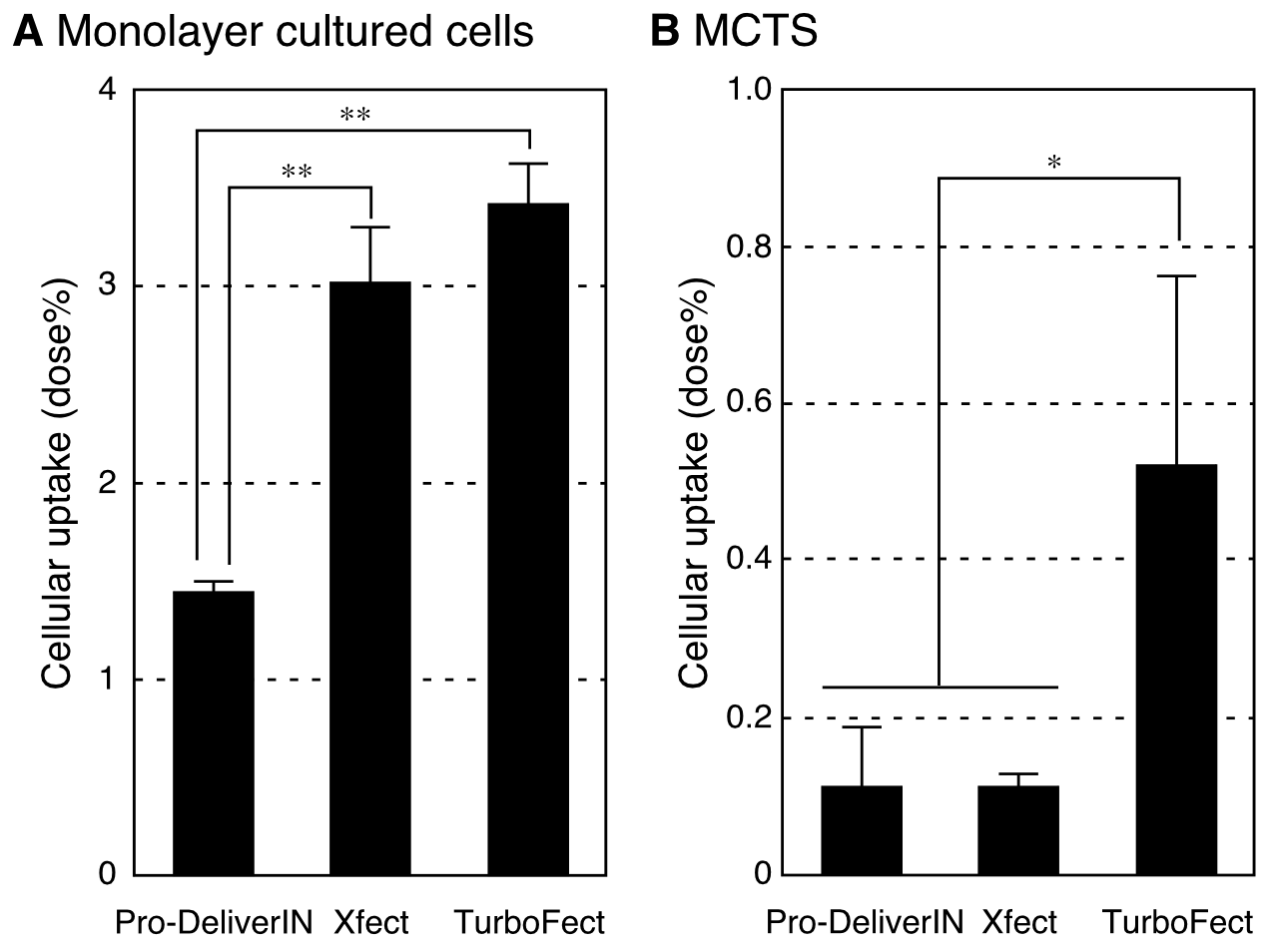
Cellular uptake of each protein transfection reagent/FITC-BSA complex. FITC-BSA complexes with Pro-DeliverIN, Xfect, and TurboFect were applied to Huh-7 monolayer cultured cells (A) and MCTS (B). Error bars in the graph represent the standard deviation, n = 4. *p<0.05 and **p<0.01. In the case of monolayer cultured cells (A), TurboFect and Xfect achieved higher cellular uptake efficiency than that of Pro-DeliverIN. MCTS experiments revealed the higher uptake of TurboFect than of Pro-DeliverIN and Xfect. The uptake of protein transfection reagents was markedly higher by monolayer cultured cells than by MCTS.

The cellular uptake of protein transfection reagents into Huh-7 monolayer cultured cells and MCTS was evaluated using FITC-conjugated BSA-incorporated complexes (Figure 1). Cell lysate treated with naked FITC-BSA showed no fluorescence in this experiment (data not shown). In the case of experiments using monolayer cultured cells, the cellular uptake of Xfect and TurboFect was significantly higher than that of Pro-DeliverIN (Figure 1A). Note that cells treated with complexes in medium without fetal bovine serum showed similar results (data not shown). The amounts of cellular uptaken FITC-BSA (dose%) by Pro-DeliverIN, Xfect, and TurboFect were 1.5, 3.0, and 3.5%, respectively. On the other hand, MCTS treated with TurboFect was taken up more efficiently than those with Pro-DeliverIN and Xfect (Figure 1B). The amounts of cellular uptaken FITC-BSA (dose%) by MCTS were markedly less than that by monolayer cultured cells, in which Pro-DeliverIN, Xfect, and TurboFect were 0.11, 0.11, and 0.52 dose%, respectively.

### Effect of endocytosis inhibitors on the internalization of BSA complexes

**Figure 2:**
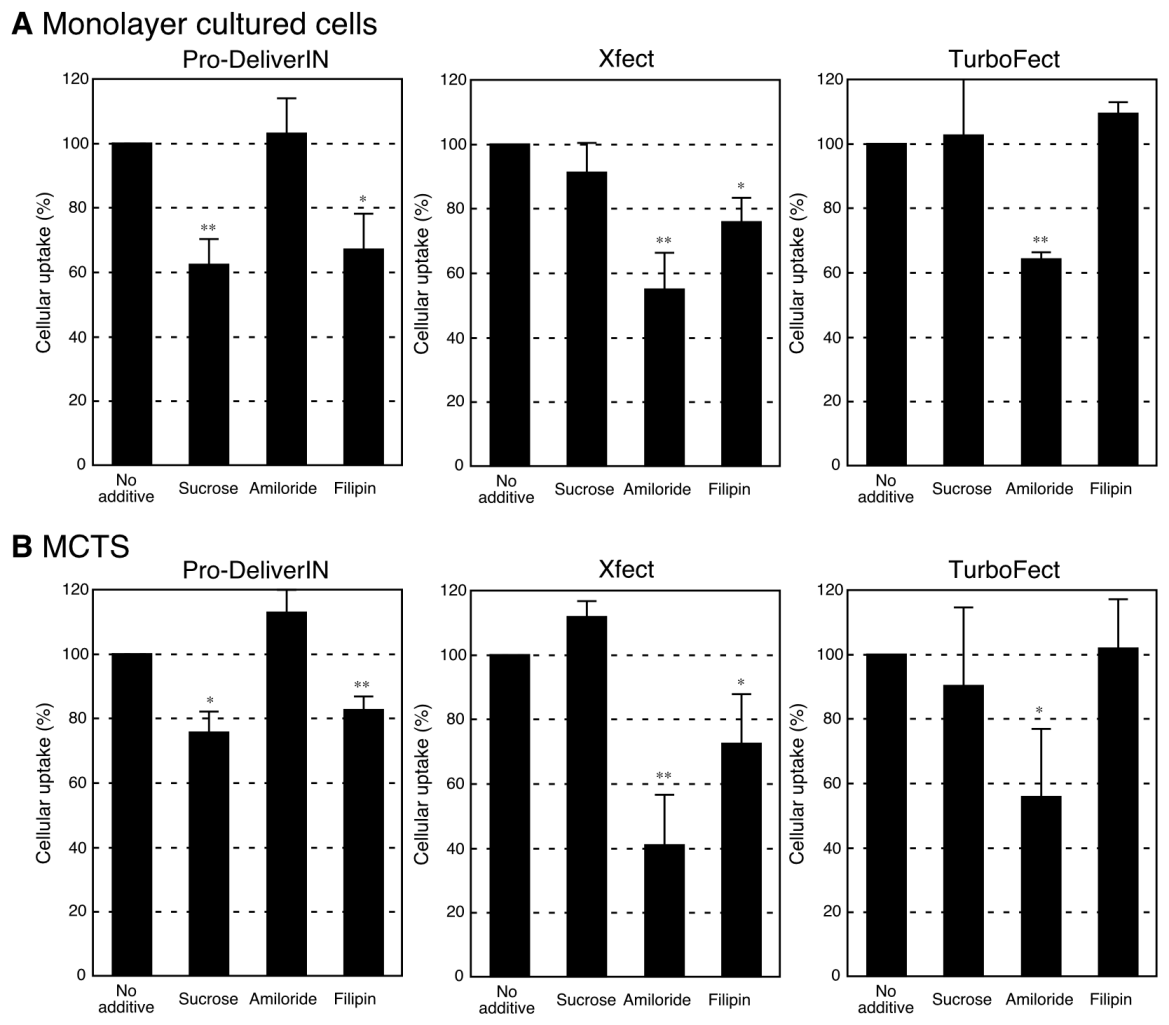
Inhibition of endocytosis using specific inhibitors. Effects of inhibitors (sucrose: clathrin-mediated endocytosis inhibitor; amiloride: macropinocytosis inhibitor; filipin: caveolae-mediated endocytosis inhibitor) on the internalization of FITC-BSA complexes with Pro-DeliverIN, Xfect, and TurboFect were evaluated using Huh-7 monolayer cultured cells (A) and MCTS (B). Error bars in the graph represent the standard deviation, n = 3 (A) or 4 (B). *p<0.05 and **p<0.01. The results obtained from monolayer cultured cells (A) and MCTS (B) were similar. Pro-DeliverIN appeared to be internalized into Huh-7 cells by clathrin-mediated endocytosis and caveolae-mediated endocytosis. The uptake route of Xfect was mainly by macropinocytosis, but also slightly by caveolae-mediated endocytosis. TurboFect was preferentially internalized into Huh-7 cells through macropinocytosis.

To clarify the mechanism of each complex internalization into Huh-7 monolayer cultured cells and MCTS, inhibitory experiments of cellular uptake were carried out using specific endocytosis inhibitors (Figure 2). The effects of the following endocytosis inhibitors on the internalization of complexes were examined: sucrose (a specific inhibitor of clathrin-mediated endocytosis); amiloride (a specific inhibitor of macropinocytosis); and filipin (a specific inhibitor of caveolae-mediated endocytosis) [14–16]. Similar results were obtained from experiments using monolayer cultured cells and MCTS. The cellular uptake of Pro-DeliverIN complexes was significantly lowered by treatments with sucrose and filipin than by the treatment with no additive control, which implied that the internalization of Pro-DeliverIN complexes into Huh-7 cells occurred via clathrin-mediated endocytosis and caveolae-mediated endocytosis. In the case of Xfect complexes, the amiloride treatment caused an approximately 40-50% decrease in the amount of cellular uptake. Filipin also significantly decreased the internalization of Xfect complexes. Xfect complexes may be mainly internalized into Huh-7 cells via macropinocytosis, but with the addition of caveolae-mediated endocytosis. The results obtained on the internalization of TurboFect complexes demonstrated that incubation in the presence of amiloride significantly affected cellular uptake, while that in the presence of sucrose and filipin did not. These results indicate that the TuboFect complexes were internalized into Huh-7 cells preferentially through macropinocytosis. It is worth mentioning again that each transfection reagent appeared to be internalized into Huh-7 monolayer cultured cells and MCTS by same mechanism. 

### MCTS distribution of complexes

**Figure 3:**
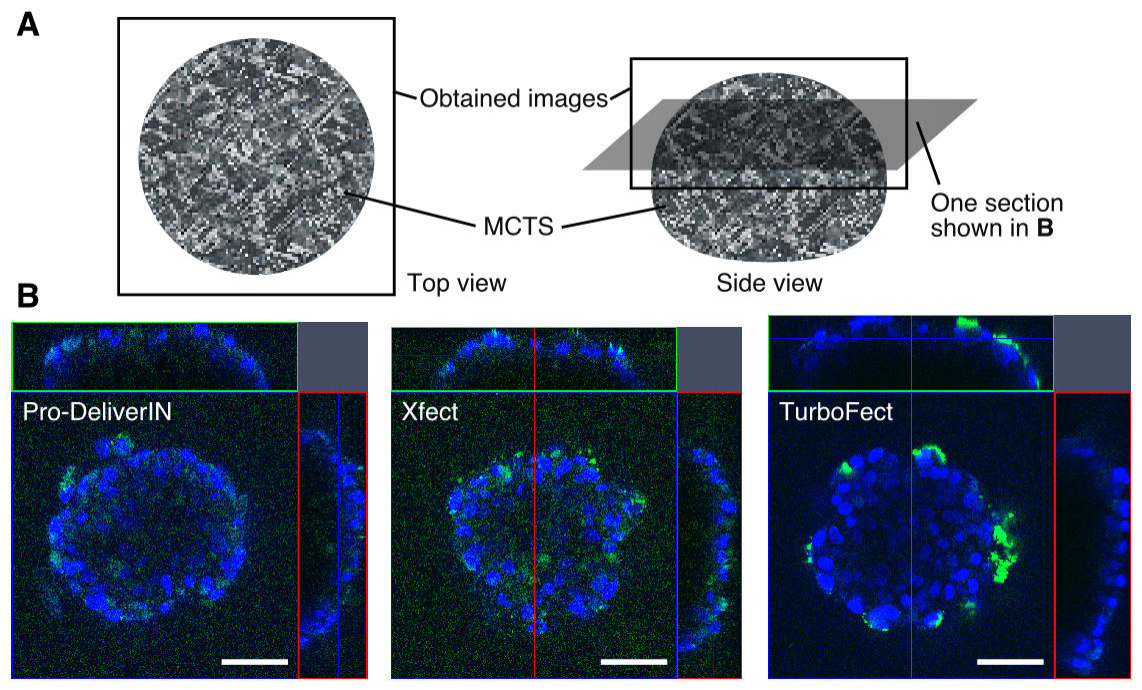
CLSM observations (40X objective) of each FITC-BSA complex using MCTS. A: Schematic illustration of MCTS observed in these experiments. The full size of MCTS was observed from the top view with the Z-stack mode from the top to middle positions. The optical thickness and the distance of the confocal section from the top of MCTS were 0.60 μm and 20 μm, respectively. B: The intra-MCTS distribution of FITC-BSA (green) complexes with Pro-DeliverIN, Xfect, and TurboFect was observed by CLSM using the 40X objective with staining nuclei (blue) shown in A. A certain amount of FITC-BSA complexes with Pro-DeliverIN and Xfect was observed in the inner layered cells. On the other hand, TurboFect showed FITC-BSA distribution only in the surface-first layered cells. The scale bars represent 50 μm.

**Figure 4:**
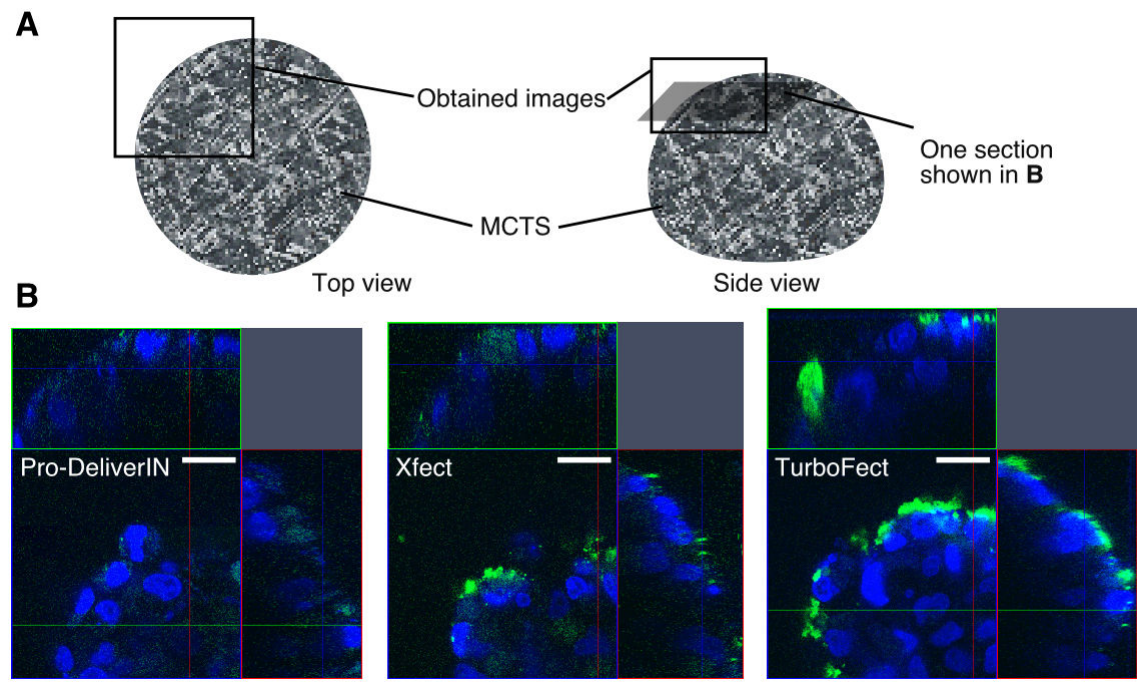
CLSM observations (100X objective) of each FITC-BSA complex using MCTS. A: Schematic illustration of MCTS observed in these experiments. About one quater size of MCTS was observed from the top view. The optical thickness and the distance of the confocal section from the top of MCTS were 0.38 μm and 20 μm, respectively. B: The intra-MCTS distribution of FITC-BSA (green) complexes with Pro-DeliverIN, Xfect, and TurboFect was observed in detail by CLSM using the 100X objective with staining nuclei (blue). A small amount of FITC-BSA was observed in the nuclei, especially in TurboFect. The scale bars represent 20 μm.

The distribution of each complex in MCTS was investigated by CLSM equipped with 40X (Figure 3) and 100X objectives (Figure 4) using FITC-BSA (green)-incorporated complexes. Hoechst 33342 was used to label nuclei (blue). Using the 40X objective, the entire spheroid was observed from the top view with the Z-stack mode from the top to middle positions (Figure 3A). The intensity of the observed FITC-BSA in MCTS treated with TurboFect complexes was the highest. These results were consistent with those of cellular uptake in Figure 1B. MCTS treated with Pro-DeliverIN and Xfect complexes showed similar distribution profiles to FITC-BSA, in which green fluorescence was mainly observed in the surface-first layered cells, but was also moderately observed in the inner layered cells. On the other hand, TurboFect complexes displayed FITC-BSA distribution only in the surface-first layered cells. A more detailed observation of FITC-BSA in MCTS was carried out in experiments using the 100X objective (Figure 4). The distribution of FITC-BSA was confirmed not only in the intracellular compartments, but also in the nuclei especially in MCTS treated with TurboFect complexes. A small amount of FITC-BSA in the Pro-DeliverIN and Xfect complexes was observed in the inner layered cells of MCTS. MCTS treated with TurboFect complexes showed green fluorescence only in the surface-first layered cells. These results were consistent with the images taken by the 40X objective. 

## Discussion

Protein therapy is increasingly recognized as a promising therapy for many intractable diseases [17,18]. Many clinical trials exploring protein therapy are currently underway and several systems are available for clinical use [19,20]. Most clinically used protein drugs have their functional roles in extracellular milieu because their target molecules are generally cell-surface receptors [21]. A next-generation protein drug may target intracellular molecules; therefore, a major key to success is the development of protein delivery systems that have high delivery efficiency not only to the target tissue, but also to the target intracellular compartment [10–13]. Several types of protein transfection reagents are commercially available for biochemical experiments [12,13,22]. They may not be available for clinical use, but it is important for researchers to know their biological properties, which may lead to the discovery of an excellent protein drug. Recent *in vitro* studies revealed that three different commercially available protein transfection reagents, Pro-DeliverIN, Xfect, and TurboFect were uptaken by monolayer cultured HeLa cells through different routes [14]. To obtain further information, we herein evaluated their protein trasfection reagents using Huh-7 MCTS models, which are useful three dimensional *in vitro* avascular tumor models.

The size and zeta-potential of BSA complexes were measured to know their physicochemical properties (Table 1). In general, polyion complexes between cationic polymers, liposomes, or peptides and anionic macromolecules such as DNA, RNA, or proteins, have positive surfaces with several tens to several hundreds nm. Contrary to our expectation, Pro-DeliverIN and TurboFect complexes had negative surface. We estimated that three commercially available reagents might include the additives, which affected the physicochemical properties of their BSA complexes.

The amount of TuboFect/FITC-BSA complexes internalized into Huh-7 MCTS was significantly higher than that of Pro-DeliverIN and Xfect complexes (Figure 1B). The results obtained from experiments using monolayer cultured cells showed that the cellular uptake of Xfect and TurboFect was more efficient than that of Pro-DeliverIN (Figure 1A). The amount of FITC-BSA (dose%) uptaken by MCTS was approximately 1/7 to 1/25 that of monolayer cultured cells, which may have been due to the number of cells used in each experiment. Five hundred Huh-7 cells were used in one experiment to evaluate MCTS and 10,000 cells were used in an experiment using monolayer cultured cells. Furthermore, the cell surface area of MCTS exposed to the medium with the complexes was much smaller than that of monolayer cultured cells even though the same number of cells were used. No cytotoxicity against both monolayer cultured cells and MCTS was observed under all experimental conditions (data not shown).

Inhibitory experiments using specific inhibitors of endocytosis were carried out in order to elucidate the intracellular internalization routes of each complex against monolayer cultured cells and MCTS (Figure 2). The results obtained from MCTS were similar to those from monolayer cultured cells, which were also consistent with previous results using monolayer cultured HeLa cells. Each protein transfection reagent may be internalized into cells through the same routes in spite of the cell type (Huh-7 and HeLa cells) [14] and cell morphology (monolayer cultured cells and spheroid cells). Pro-DeliverIN complexes were preferentially internalized into Huh-7 cells via clathrin-mediated endocytosis and caveolae-mediated endocytosis. Huh-7 cells took up Xfect complexes by macropinocytosis and, in addition, caveolae-mediated endocytosis. On the other hand, only macropinocytosis appeared to be the internalization route of TuboFect complexes. Based on these results, we can choose and use an appropriate protein transfection reagent according to the type of experiment being conducted.

CLSM observations with nuclei staining (blue) were carried out to clarify the distribution of each complex in MCTS (Figures 3 and 4). Figure 3B shows each complex distribution (green) in MCTS using the 40X objective. TuboFect complexes were distributed only in the surface-first layered cells. MCTS treated with Pro-DeliverIN and Xfect complexes showed strong FITC-BSA distribution in the surface-first layered cells, but modest distribution in the inner layered cells. Figure S1 showed the results of semi-quantification of fluorescence intensity in the first layered cells or second layered cells. Fluorescence intensity of TuboFect complexes in the second layered cells were much smaller than those in the first layered cells, which is consistent with the results in Figures 3 and 4. Two possibilities have been proposed for the appearance of green color in the inner cells of MCTS. The first is that complexes went through intercellular compartments, in which the extracellular matrix including polysaccharides and proteins existed. It is generally difficult for macromolecules and nano-sized particles to move freely across the extracellular matrix [2]. The second is that transcytosis caused percolation of the complexes. A similarity between Pro-DeliverIN and Xfect complexes is that caveolae-mediated endocytosis was involved in Huh-7 cellular uptake. In addition, caveolae-mediated transcytosis has been reported to be a possible vesicular trafficking pathway through cell barriers [23,24]. Thus, it is reasonable to assume that some Pro-DeliverIN and Xfect complexes could pass through the surface-first layered cells by caveolae-mediated transcytosis. The results obtained from CLSM observations using specific endocytosis inhibitors were consistent with this hypothesis (Figure S2). Filipn, which is a caveolae-mediated endocytosis inhibitor, obviously affected on the percolation of Pro-DeliverIN and Xfect complexes, while sucrose and amiloride did not. CLSM observations using the 100X objective revealed a more detailed complex distribution in MCTS, in which small amount of complexes was colocalized in the nuclei of surface-first layered cells (Figure 4B). The outer cell layers of MCTS actively proliferate, which may allow the complexes to enter nuclei during mitosis. CLSM observations were not performed completely because it was difficult to observe deep inner layered cells in MCTS [7,8]. However, the results obtained here exhibited a clear decline according to the each reagent, even in the observations of surface-second layered cells in MCTS.

In conclusion, three commercially available protein transfection reagents, Pro-DeliverIN, Xfect, and TurboFect were evaluated using Huh-7 cells in this study. Each reagent had preferential cellular uptake routes, which were the same by monolayer cultured cells and MCTS of Huh-7. In addition, the internalization mechanism affected the distribution of each reagent in MCTS, that is, caveolae-mediated endocytosis/transcytosis delivered Pro-DeliverIN and Xfect complexes to the inner cells. These results may be helpful in choosing and using protein transfection reagents for *in vitro* monolayer cultured cells and MCTS experiments with the aim of evaluating anti-tumor therapeutic proteins.

## Supporting Information

Figure S1 **Semi-quantitative analyses of intra-MCTS distribution of FITC-BSA complexes.** Fluorescence intensity of each first layered cell or second layered cell was measured from the obtained images by CLSM using the 40X objective. Error bars in the graph represent the standard deviation, n = 14. *p<0.05 and **p<0.01.
(EPS)

Figure S2 **CLSM observation of each complex using MCTS with specific endocytosis inhibitors.** Effects of inhibitors on the internalization of FITC-BSA complexes were evaluated using Huh-7 MCTS by CLSM observation (40X objective). The optical thickness and the distance of the confocal section from the top of MCTS were 0.60 μm and 20 μm, respectively. A: MCTS was observed in the absence or presence of sucrose (a clathrin-mediated endocytosis inhibitor) under the condition of 30 min pre-incubation and 3h incubation of FITC-BSA complexes. B: MCTS was observed in the absence or presence of amiloride (a macropinocytosis inhibitor) or filipin (a caveolae-mediated endocytosis inhibitor) under the condition of 30 min pre-incubation and 1h incubation of FITC-BSA complexes. The scale bars represent 50 μm.
(EPS)
